# Natural *Trypanosoma cruzi* Infection and Climatic Season Influence the Developmental Capacity in Field-Caught *Mepraia spinolai* Nymphs

**DOI:** 10.3390/insects14030272

**Published:** 2023-03-09

**Authors:** Juan Botzotz, Gabriel Méndez-Valdés, Sylvia Ortiz, Angélica López, Carezza Botto-Mahan, Aldo Solari

**Affiliations:** 1 Programa de Biología Celular y Molecular, Instituto de Ciencias Biomédicas, Facultad de Medicina, Universidad de Chile, Santiago 8380000, Chile; 2 Departamento de Ciencias Ecológicas, Facultad de Ciencias, Universidad de Chile, Santiago 7800003, Chile

**Keywords:** *Mepraia spinolai*, *Trypanosoma cruzi*, instar-dependent molting, sylvatic kissing bug, Chagas disease vector, molting efficiency, Chile

## Abstract

**Simple Summary:**

Triatomines are hemimetabolous and hematophagous insect vectors of *Trypanosoma cruzi*, the parasite that causes Chagas disease. The life cycle of triatomines is composed of five nymphal stages and an adult stage. The wild kissing bug *Mepraia spinolai* is distributed in a vast area of the semiarid Mediterranean ecosystem of South America, characterized by strongly marked seasons. The population structure of this vector changes during the different seasons, suggesting its development is controlled under natural conditions. In this study, we performed a biological assay to study this vector development after two laboratory feedings. We collected nymphs, first to fourth instars, in eight *M. spinolai* populations during the transition of late fall to winter (cooling period) and spring (warming period) of three consecutive years. The gathered information with vectors molting twice suggests that development is differentially controlled in nymphs from first and fourth instars depending on the climatic period of collection. Additionally, *T. cruzi* infection changes development forward only in nymphs of second and fourth instars of the warming period. Our findings suggest that the effect of the climatic period and *T. cruzi* infection on the development of this vector species is a stage-dependent phenomenon.

**Abstract:**

In this study, we evaluated the effect of the climatic season and infection by *Trypanosoma cruzi*, etiological agent of Chagas disease, on the molting capacity of the triatomine vector *Mepraia spinolai* endemic to Chile. We used wild-caught first-to-fourth instar nymphs during cooling (fall and winter) and warming (spring) periods. After capturing, nymphs were fed at the laboratory, and maintained under optimal rearing conditions. Feeding was repeated 40 days later. We followed-up the molting events on 709 nymphs, recording one, two or the absence of molts after two feeding opportunities. Within the same climatic period, only infected second- and fourth-instar nymphs from the warming period showed a larger proportion of double molting compared to uninfected nymphs. Regarding the climatic period, infected and uninfected first- and fourth-instar nymphs exhibited a larger proportion of double molting in the warming and cooling periods, respectively. The pattern of non-molting nymph occurrence suggests they probably reach diapause by environmental stochasticity. The effect of the climatic period and *T. cruzi* infection on the development of *M. spinolai* is an instar-dependent phenomenon, highlighting the occurrence of finely synchronized processes at different moments of the life cycle of such an hemimetabolous insect as triatomines.

## 1. Introduction

Triatomine bugs (Hemiptera: Reduviidae) are hemimetabolous and hematophagous insects distributed in the Americas, capable of transmitting *Trypanosoma cruzi*, the etiological agent of Chagas disease [1]. Triatomine development includes five nymphal stages and an adult stage. Several demographic factors can modify triatomine population dynamics such as age structure, life history trait variation caused by environmental fluctuations, dispersal in spatially heterogeneous environments, and local extinction and colonization, as well as *T. cruzi* infection [2,3,4,5,6,7,8,9].

In vector-borne infections, vectors can also be considered as an alternative host for the parasite; therefore, some degree of vector exploitation is to be expected [10,11]. In the case of *T. cruzi*, the pathogenicity to the insect vector can be gauged via mortality, reproduction, and developmental arrests (diapause). In nature, developmental delays are observed in triatomines submitted to different conditions such as low environmental temperature [3], density-dependent access to blood sources [12], or diapause triggered by environmental factors such as changes in weather [7]. Almost a century ago, a study on the molting process of the triatomine *Rhodnius prolixus* was carried out, detecting that a full engorgement was necessary to trigger ecdysis [13]. Currently, it is known that the initial steps of triatomine development involve the release of the neuropeptide prothoracicotropic hormone and ecdysteroids in the hemolymph within the neuroendocrine system [14].

Experimental studies evaluating the effect of *T. cruzi* in triatomine development have shown variable results under optimal climatic conditions [15,16,17,18,19], mostly depending on the experimental design. However, experimental studies assessing the effect of *T. cruzi* on triatomine development submitted to variable abiotic conditions are less represented. For example, in *R. prolixus*, a development retardment was observed in *T. cruzi*-infected insects and the molting time was shorter at 27–30 °C than at lower temperatures [11]. Regarding the effect of environmental temperature on the development of uninfected *Triatoma brasiliensis*, it was shown that no ecdysis occurred between 12 °C and 21 °C [20]. Few studies on the development of triatomines submitted to natural climatic conditions are available. Laboratory-reared *Triatoma infestans* studied in the field under the variable environmental conditions of the austral winter of Argentina detected developmental arrests in nymphs of all instars [3]. Similarly, studies on laboratory-reared *T. infestans* submitted to temperatures and photoperiods found that under natural conditions, feeding ceases below 16–18 °C; the same is observed for reproduction [21,22]. Insects might regulate development forward, causing molting arrests or changing vital rates that translate into different ratios of developmental stages during the life cycle [7,23,24]. The developmental response of holometabolous insects to the photoperiod length has been studied, detecting regulations of seasonal morphs, growth rates, and diapause, among others [24,25,26]. Notwithstanding, no information has been reported on how and when development regulations are controlled in triatomine bugs (hemimetabolous insects), depending on climatic conditions.

*Mepraia spinolai* is considered the most epidemiologically important triatomine species in Chile due to its wide distribution, 26°–34° S, from the coast up to 3000 m.a.s.l. [27], an area with four marked climatic seasons [28], and the high frequency of *T. cruzi* infection in its populations [29,30,31]. This triatomine species mainly use stockpiles and bromeliads as refuge, where it coexists with small mammals and reptiles; both groups of vertebrates are included in its diet [32,33,34] and are part of the sylvatic transmission of *T. cruzi* in the semiarid Mediterranean ecosystem [35,36,37,38,39]. In addition, wild-caught *M. spinolai* individuals exhibit blood stealing between conspecifics, increasing the complexity of *T. cruzi* transmission under natural conditions [40].

In nature, *M. spinolai* populations exhibit a spatially patchy distribution with variable densities and a population structure of predominantly early-instar nymphs in late austral summer–fall, and one represented by all developmental stages in austral spring–early summer [29,41]. Adult females of this species are macropterous and adult males present three alary morphs: macropterous, brachypterous, and macropterous; mostly macropterous males invade human dwellings [42]. Reproduction occurs from late austral spring to early fall, when adults are present. During the warm seasons, the home range of *M. spinolai* colonies is larger and their blood sources are more diverse than in the cold seasons [32,43]. Under optimal laboratory rearing conditions, the generation time of *M. spinolai* varies from 9 to 10 months, while in nature—characterized by high thermal oscillation and variable photoperiods—it takes up to 12 months to molt to the fifth instar [19,44,45].

In this study, we examined the developmental process (molting events)—under optimal laboratory-controlled conditions (27 °C; 70% relative humidity (RH))—of wild-caught *M. spinolai* nymphs (first to fourth instars) either naturally infected with *T. cruzi* or uninfected. Nymphs were collected in late fall and winter (i.e., cooling period) and early and late spring (i.e., warming period) over three consecutive years (2016–2018). To this end, nymphs were fed ad libitum twice after capture to evaluate molting capacity. The goals of our study were: (i) to determine how natural infection with *T. cruzi* affects the molting capacity of *M. spinolai* nymphs of various instars collected in cooling and warming periods; and (ii) to determine if *M. spinolai* nymphs collected in cooling and warming periods regulate development at a particular nymphal stage.

## 2. Materials and Methods

### 2.1. Study Area and Triatomine Collection

**Figure 1:**
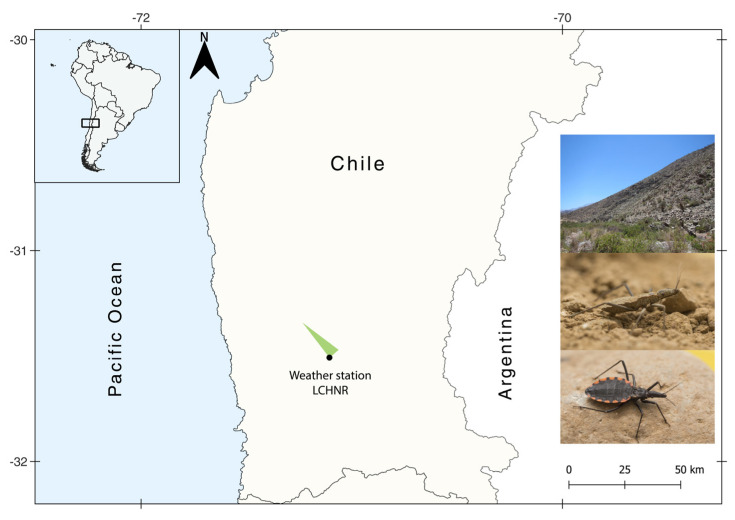
Map of Chile, South America, showing the area of *Mepraia spinolai* collection and the location of the Weather Station at Las Chinchillas National Reserve, CONAF, Coquimbo Region. On the right side: view of a collecting site (**top**), *M. spinolai* nymph (**middle**), and adult female (**bottom**).

Triatomine nymphs were collected from three localities (Las Chinchillas National Reserve, Los Pozos, and El Cuyano; Coquimbo Region, north-central Chile), covering a total area of 61.5 km^2^ (Figure 1). *M. spinolai* populations, defined as a group of bugs captured in an area of approximately 50 m^2^ within a specific period of time, were collected from different north-facing slopes in the area. The area is a hyper-endemic zone of Chagas disease, with *M. spinolai* the most abundant triatomine species, and characterized by a semiarid Mediterranean climate with rocky outcrops and scarce vegetation cover, mostly represented by bromeliads (*Puya* sp.) and cacti. Domestic (goats, cats, dogs, sheep), native (rodents, marsupials, foxes, bats), free-ranging introduced (European rabbits) mammals and lizard species can be found in this area, with several of these vertebrate species acting as blood meal of *M. spinolai* and *T. cruzi* reservoirs [30,34,35,38,39].

Triatomine collection was manually performed by one trained researcher over the course of one week in the cooling period and over the course of three days in the warming period, from 11:00 to 16:00 h in rocky outcrops, the period of maximum activity [46]. Triatomines were collected from rocks as they came closer to the researcher (i.e., without removing the rocks). Captured bugs were transported to the laboratory in polystyrene foam boxes, with folded papers as refuge, within one to two days after capture, and kept individually in plastic boxes in a climatic chamber at 27 °C, 70% RH, and a 14:10 h light:dark photoperiod until feeding.

### 2.2. Triatomine Fecal Sample Collection

Within the next two weeks, nymphs were fed with uninfected mice (*Mus musculus*) anesthetized with 2% sodium pentothal (Laboratorios RICHMOND, Buenos Aires, Argentina). Mice of 2 months of age were obtained from the vivarium of the Faculty of Medicine, University of Chile. Procedures of animal handling were performed according to the rules and with the permission of the Animal Ethics Committee of the University of Chile (CBA#0987-FMUCH-2016, CBA#1027-FMUCH-2019). After feeding, each nymph was classified by nymphal stage and kept separately in a plastic container with small compartments of 3.2 × 3.6 cm to allow for individual follow-up. Fecal samples were collected 30 min after feeding without pressing the abdomen to avoid internal damage, i.e., as a result of spontaneous defecation, and mixed with 100 μL of distilled water. This procedure was repeated for each nymph after a second feeding 40–45 days later. Nymphs were maintained in a growth chamber under the same conditions previously described. Death and ecdysis were recorded after each feeding. Only fed nymphs visually engorged and surviving the two feeding events were used for further analysis.

### 2.3. DNA Extraction and T. cruzi Infection Detection

Fecal samples were individually extracted in a final volume of 200 μL, using conditions already described by manufacturers of the EZNA Blood DNA kit (OMEGA BIO-TEK, Norcross, GA, USA). *T. cruzi* presence was tested three times by PCR analysis directed to minicircle kDNA with oligos 121 and 122, using different volumes of extracted DNA as templates in a final volume of 50 μL to improve detection [47,48]. A volume of 10 μL of each reaction mixture was run in agarose gel electrophoresis (Lafken, Santiago, Chile) and stained with ethidium bromide (Invitrogen, Waltham, MA, USA). Each PCR assay was repeated when negative results were obtained. In those samples, the extracted DNA was concentrated three times by evaporation, and then three new PCR assays were repeated using variable volumes of the concentrated DNA as a template. A negative sample resulted when all PCR assays failed to detect a 330 bp amplicon. This kind of amplicon (i.e., pattern of bands) is characteristic of *T. cruzi* minicircles, ensuring the amplification is not *Trypanosoma rangeli*, which also amplify with this couple of primers [49].

### 2.4. Statistical Analysis

Abiotic variables (temperatures and photoperiod) from cooling and warming periods were compared with *t*-tests. The proportion of *T. cruzi*-infected nymphs (all instars combined) from the cooling and warming periods were compared by χ^2^ tests, as well as the proportion of nymphs (all instars combined) molting twice (after two feeding events), depending on the infection status within each climatic period and vice versa (i.e., depending on the climatic period, keeping infection status constant).

For each developmental stage (first to fourth), the association between double molting and *T. cruzi* infection was tested by χ^2^ tests within the same climatic period (cooling or warming). The same test was used to evaluate the association between double molting and climatic period—for each developmental stage—within the same infection status (uninfected or *T. cruzi*-infected). The same test was used to examine the association between complete absence of molting (after two feeding events) and infection status (or climatic period), but considering all developmental stages combined due to the low number of non-molting nymphs. The significance level (or alpha level) considered statistically significant in this study was 0.05. Analyses were performed with R (version 3.6.0, R Development Core Team 2019) or JMP-Pro (version 14) (SAS Institute Inc., Cary, NC, USA)

## 3. Results

### 3.1. M. spinolai Populations and Climatic Conditions

Between 2016 and 2018, a total of eight *M. spinolai* populations were sampled, four in each climatic period (cooling: late fall and winter; warming: spring). A total of 343 and 366 nymphs were collected in the cooling and warming periods, respectively. Details on each *M. spinolai* population used in this study (period, date of capturing, total number of captures) and abiotic conditions (photoperiod, minimum and maximum temperatures one week prior to collection events) are summarized in Table 1. Only the mean maximum temperatures showed statistically significant difference between warming and cooling periods (mean min T° ± SE: cooling = 5.00 ± 0.42, warming = 5.30 ± 0.36, t_(54)_ = −0.59, *p* = 0.280; mean max T° ± SE: cooling = 20.86 ± 0.62, warming: 23.23 ± 0.53, t_(54)_ = −2.90, *p* = 0.003). As expected, the warming period presented, on average, more daylight hours than the cooling period (mean daylight hours ± SE: cooling = 10.30 ± 0.12, warming = 12.75 ± 0.46, t_(6)_ = −5.18, *p* = 0.001). The complete dataset of minimum and maximum temperatures is available in Table S1.

### 3.2. Molting Events: Climatic Period and T. cruzi Infection

Overall, more than half of nymphs (all instars and infection status combined) from the cooling and warming periods molted only once, 63.9% and 60.9% after two feeding events, respectively. Only 27.4% and 31.4% of the nymphs from cooling and warming periods were capable of molting twice under the same conditions, respectively. Less than 10% of the nymphs were unable to molt (8.8% in cooling and 7.7% in warming). Details (of numbers) of nymphs by developmental stage (first to fourth) and type of molting (one, twice, none) in each *M. spinolai* population are shown in Table 2.

Regarding *T. cruzi* infection, nymphs (all instars combined) from the warming period were twice more infected with *T. cruzi* than those from the cooling period (56.8% vs. 21.9%, respectively; χ^2^ = 139.81, *p* < 0.0001). Within the warming period, a larger proportion of infected nymphs molted twice than those uninfected (36.1% vs. 25.3%, respectively; χ^2^ = 4.81, *p* = 0.028), but this molting behavior was not detected in nymphs from the cooling period (33.3% vs. 25.7%, respectively; χ^2^ = 1.70, *p* = 0.193). Altogether (combining all developmental stages from the same climatic period by infection status), regardless of the infection status, we did not detect statistically significant differences between cooling and warming periods in the proportion of nymphs exhibiting double molting (infected: 33.3% vs. 36.1%, respectively, χ^2^ = 0.1791, *p* = 0.672; uninfected: 25.7% vs. 25.3%, respectively, χ^2^ = 0.01, *p* = 0.922). Details on total number of nymphs by developmental stage (first to fourth instar), type of molting (one, twice, none), and *T. cruzi* infection combining populations from the same climatic period (cooling and warming) are summarized in Table 3. The complete dataset at the individual basis is available in Table S1.

### 3.3. Nymphs Molting Twice: Developmental Stage, T. cruzi Infection, and Climatic Period

**Figure 2:**
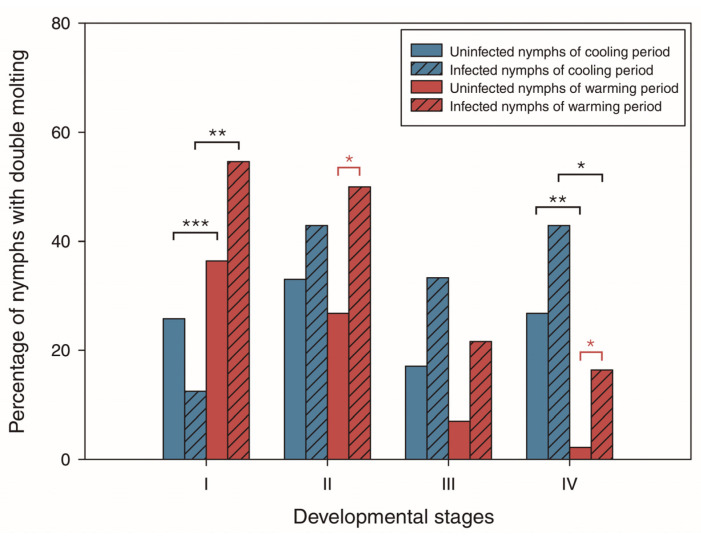
Percentage of *Trypanosoma cruzi*-infected and uninfected *Mepraia spinolai* nymphs molting twice after two feeding events, combining the same developmental stage of populations from the cooling or warming periods for the three years of collection (2016–2018). Blue and red bars represent nymphs from the cooling and warming periods, respectively. Hatched bars represent *T. cruzi*-infected nymphs. Black contrasts represent comparisons between nymphs of different periods but with the same infection status. Red contrasts represent comparisons between nymphs of different infection status within the same climatic period. Only statistically significant contrasts are shown. Significance levels: * < 0.05, ** < 0.01, *** < 0.001.

Overall, 209 nymphs (first to fourth instars) molted twice after the two feeding events (94 from cooling and 115 from warming). Within the same climatic period, only infected second- and fourth-instar nymphs from the warming period showed a larger proportion of double molting compared to uninfected nymphs of the same instar (second instar: 50.0% vs. 26.8%, respectively, χ^2^ = 5.61, *p* = 0.018; fourth instar: 16.4% vs. 2.2%, respectively; χ^2^ = 5.59, *p* = 0.018; Figure 2). No nymphs of the remaining instars from the warming (first and third) and cooling (first to fourth) periods showed statistically significant differences between the probability of double molting depending on the infection status (*p* > 0.05).

Separated analyses by developmental stages showed that first-instar nymphs, regardless of their infection status, exhibited a larger proportion of double molting in the warming period compared to those from the cooling period (infected: 54.6% vs. 12.5%, respectively, χ^2^ = 8.45, *p* = 0.004; uninfected: 59.1% vs. 25.8%, respectively, χ^2^ = 11.92, *p* < 0.001; Figure 2). On the other hand, fourth-instar nymphs—regardless of their infection status—exhibited a larger proportion of double molting in the cooling period compared to those from the warming period (infected: 42.9% vs. 16.4%, respectively, χ^2^ = 4.75, *p* = 0.029; uninfected: 26.8% vs. 2.2%, respectively, χ^2^ = 10.82, *p* = 0.001; Figure 2). No other infected or uninfected nymphs of remaining instars (second and third) showed statistically significant differences between the probability of double molting depending on climatic period of collection (*p* > 0.05).

### 3.4. Non-Molting Nymphs: Infection and Climatic Period

Overall, 58 nymphs across all developmental stages did not molt after the two feeding events, 30 and 28 from the cooling and warming periods, respectively (Table 2). Within the same climatic period, we did not detect differences between infected and uninfected nymphs in their probability of not molting (cooling: 6.7% vs. 9.6%, respectively, χ^2^ = 0.63, *p* = 0.427; warming: 6.7% vs. 8.9%, respectively, χ^2^ = 0.58, *p* = 0.448; Table 3). Within the same infection status, we did not detect differences between nymphs from cooling and warming periods in their probability of not molting (infected: 6.7% for both climatic periods, χ^2^ = 0.0004, *p* = 0.95; uninfected: 9.6% vs. 8.9%, respectively, χ^2^ = 0.07, *p* = 0.792; Table 3). Because we did not detect differences by infection and collecting period in the frequency of non-molting nymphs, we tested for differences among nymphal stages (combining both infection status and periods). We detected overall differences among the frequency of non-molting nymphs depending on the nymphal stage (first: 2.4%; second: 6.2%; third: 12.6%; fourth: 12.4%; χ^2^ = 16.30, *p* = 0.001; Table 2).

## 4. Discussion

In this study, we have designed a simple biological test to study the development of field triatomines. Previously, we have performed follow-up studies in wild-caught *M. spinolai* after successive feedings to test the effect of *T. cruzi* infection on insect development and survivorship [50,51,52]. The gathered results from the mentioned studies showed that all nymphal stages are able to molt under laboratory-standard conditions. In the present study, nymphs were fed twice (the first feeding right after capture) and maintained under optimal abiotic conditions; therefore, any detected effect in the development should mostly depend on biotic or abiotic factors the insect experienced in the field. The results of first-to-fourth instar nymph development after the two feeding events generated three kinds of molting patterns: nymphs molting once (N = 442; 62.3%), molting twice (N = 209; 29.5%), or not molting at all (N = 58; 8.2%). This result suggests that *M. spinolai* nymphs are heterogeneous in nature regarding their nutritional and physiological conditions. The first group is split into two subgroups of insects, those molting after the first feeding and those after the second feeding. Previous studies using field triatomines in other latitudes have revealed that blood meals are often difficult to obtain and starvation seems to be frequent [53,54]. However, the mechanisms underlying our results of one-molt events are not straightforward.

Regarding the group of nymphs molting twice, these insects were more homogeneous since they probably molted with extensive abdominal distensions, after the two feeding events [55,56]. Overall, *T. cruzi*-infected nymphs (all stages combined) from the warming period presented double molting (i.e., maximum molting capacity) more frequently than equivalent uninfected nymphs. A detailed analysis for each nymphal stage revealed that second- and fourth-instar nymphs infected by *T. cruzi* presented higher molting capacity than equivalent uninfected insects, but only in those captured in the warming period. This result is surprising since this implies *T. cruzi* infection is associated with increased molting capacity, contrary to what is expected for a potentially detrimental pathogen. In fact, the experimental infection with *T. cruzi* of laboratory-reared *M. spinolai* (first generation), under optimal conditions, generated development retardment in the last three nymphal stages [19]. Other alterations in life history traits of *T. cruzi*-infected *M. spinolai* have been reported, including the shortening of defecation time, increasing biting rate, and faster approaching behavior to humans [57,58]. These results suggest that the presence of *T. cruzi* might favor parasitic transmission towards hosts [59]. The contrasting results between experimentally infected insects and naturally infected insects suggest that the origin of *M. spinolai* (laboratory-reared versus wild-caught nymphs), the type of *T. cruzi* infection (e.g., DTU identity, amount of *T. cruzi* entering with the blood meal, or frequency of infected blood meals), microbiota changes (i.e., bacteria composition after triatomine feeding), and/or the blood source are important factors that might explain, at least in part, the observed differences [60,61,62,63,64].

The higher molting capacity of second- and fourth-instar *T. cruzi*-infected nymphs found only in insects collected in the warming period is interesting. In this study, we showed that *M. spinolai* nymphs (all instars combined) exhibited higher *T. cruzi* prevalence in this period, and since insects are ectotherms, this translates to them having higher metabolic activity and mobility in the warming period. In parallel, *T. cruzi* also positively responds to higher temperatures; therefore, infected *M. spinolai* nymphs compensate the exerted *T. cruzi* exploitation by means of higher feeding efficiency (i.e., more voracious nymphs searching for more feeding opportunities, probably until engorgement), triggering more molting events and development forward [43,57,58,65,66,67].

Regarding the climatic period, nymphs did not differ between cooling and warming periods in the proportion of double molting, regardless of infection status, when all developmental stages were analyzed in combination. However, a detailed analysis separated by nymphal stage revealed that first-instar nymphs—regardless of their infection status—presented maximum molting capacity, reaching the third nymphal stage, when collected in the warming period. On the other hand, fourth-instar nymphs showed a maximal molting capacity, reaching the adult stage (i.e., incomplete metamorphosis), when collected in the cooling period. To the best of our knowledge, no other studies using field triatomines have examined the molting pattern separated by nymphal stage. These results suggest that development is controlled forward in first-instar nymphs, increasing the proportion of the next nymphal stages (e.g., second- and third-instar) in *M. spinolai* populations during warmer periods, and in the fourth-instar nymphs, increasing double molting in the cooling period, translating into adult occurrence when the warming period initiates in early spring. Adults of *M. spinolai* are observed in the field from mid-spring until late summer when the appropriate abiotic conditions for reproduction occur, and vertebrate abundance is optimal for blood meals [41,43]. In other latitudes, the population dynamics of *Triatoma dimidiata* in the field revealed positive associations of nymphs’ abundance and the presence of adults with the occurrence of the dry and hot seasons of Mexico [6]. In austral latitudes of Argentina, *T. infestans* fecundity and population numbers are associated with rising temperatures in early spring [68].

Developmental events in holometabolous insects such as egg hatching, pupation, and metamorphosis occur only once in their lives (circannual), and clock control could explain how a rhythm in such an event is also produced by a circadian clock [69]. In this line of evidence, the response to photoperiod has been identified in several insect species; it is demonstrated to be involved in growth rate regulation, migration strategy, and diapause [25]. We propose that environmental temperatures, the photoperiod, or both factors could regulate the developmental process of this hemimetabolous wild triatomine species.

In this study, a third group of nymphs are those that did not molt after either of the two feeding occasions. This is a small number of insects, prevalent neither in the warming period nor the cooling period but, interestingly, first-to-fourth-instar nymphs within the same *M. spinolai* population presented this behavior. Notwithstanding, a higher proportion of third- and fourth-instar nymphs presented molting absence compared to first- and second-instar nymphs. This result suggests that in our study, molting failure might be due to environmental stochasticity, and not to demographic stochasticity, since the latter phenomenon arises only in individuals of a given developmental stage [23]. Interestingly, three out of the five *M. spinolai* populations showing absence of molting were time-related. Specifically, C3, C4, and W4—all 2018′ populations—presented maximum rates of molting failure in a year with an unusually warm fall [48]. Therefore, our results of molting failure could be interpreted as diapause by environmental stochasticity, which seems to end after an unknown period of time after failure.

In conclusion, development is differentially controlled in nymphs from first and fourth stages depending on the climatic period of collection, and *T. cruzi* infection changes development forward only in nymphs of second and fourth instars of the warming period. Therefore, the effect of the climatic period and *T. cruzi* infection on the development of the austral kissing bug *M. spinolai* is an instar-dependent phenomenon, highlighting the occurrence of finely synchronized processes at different moments of the life cycle of such an hemimetabolous insect as this wild triatomine.

## Figures and Tables

**Table 1:** Total number of *Mepraia spinolai* nymphs (first to fourth stage) captured in each collection event that survived the two feeding procedures. Photoperiod (L:D) at the time of capturing and mean (±SE) minimum/maximum temperatures (°C) one week before collection are indicated.

Population	Period	Month/Year	Nº of Captures	L:D	Min T°	Max T°
C1	Cooling	May 2016	79	10.5:13.5	8.37 ± 0.53	21.51 ± 0.99
C2	Cooling	July 2017	60	10.1:13.9	4.34 ± 0.15	17.71 ± 0.79
C3	Cooling	June 2018	100	10.1:13.9	4.29 ± 0.22	24.00 ± 1.07
C4	Cooling	August 2018	104	10.5:13.5	2.91 ± 0.29	20.20 ± 0.50
W1	Warming	October 2016	100	12.8:11.2	5.09 ± 0.27	24.57 ± 0.91
W2	Warming	September 2017	47	11.5:12.5	3.73 ± 0.63	20.34 ± 0.71
W3	Warming	November 2017	40	13.7:10.3	7.91 ± 0.28	24.91 ± 0.76
W4	Warming	October 2018	179	13.0:11.0	4.49 ± 0.20	23.09 ± 1.02

Nº: number; T°: temperature.

**Table 2:** *Mepraia spinolai* nymphs molting once, twice, or not at all after the two feeding events for each population captured during the cooling and warming periods. Total numbers of nymphs are ordered by developmental stage (first to fourth stage; I to IV) and type of molting (one, two, or none) is shown in the same order. Total number represents the developmental stage at capturing.

			Nº Molting Events		
Population	Nymphal Stage	Total Number	One	Two	None
C1	I-II-III-IV	16-28-13-22	14-24-12-14	2-4-1-8	0-0-0-0
C2	I-II-III-IV	24-16-10-10	18-13-7-4	6-2-2-6	0-1-1-0
C3	I-II-III-IV	21-31-38-10	10-13-29-4	8-13-1-1	3-5-8-5
C4	I-II-III-IV	15-43-33-13	15-17-15-10	0-22-16-2	0-4-2-1
W1	I-II-III-IV	28-46-20-6	11-29-12-5	16-14-2-1	1-3-6-0
W2	I-II-III-IV	15-16-5-11	2-2-4-3	13-14-1-8	0-0-0-0
W3	I-II-III-IV	18-12-5-5	5-7-3-3	13-5-2-2	0-0-0-0
W4	I-II-III-IV	27-33-35-84	19-21-27-70	8-11-5-0	0-1-3-14

Nº: number; C: cooling; W: warming.

**Table 3:** Total number of *Mepraia spinolai* nymphs (first to fourth stage; I to IV) molting once, twice, or not molting after two feeding events, combining the same developmental stage of populations from the cooling or warming periods for the three years of collection (2016–2018). Parentheses indicate the number of infected nymphs within each total.

	Molting Events					
	Cooling Period			Warming Period		
Nymphal Stage	One	Two	None	One	Two	None
I	57 (14)	16 (2)	3 (0)	37 (19)	50 (24)	1 (1)
II	67 (9)	41 (9)	10 (3)	59 (31)	44 (33)	4 (2)
III	63 (15)	20 (8)	11 (1)	46 (26)	10 (8)	9 (3)
IV	32 (7)	17 (6)	6 (1)	81 (43)	11 (10)	14 (8)

## Data Availability

The data presented in this study are available in the manuscript and in its Supplementary Material (Table S1).

## Supplementary Materials

The following supporting information can be downloaded at: https://www.mdpi.com/article/10.3390/insects14030272/s1, Table S1: Climatic records from Las Chinchillas National Reserve, CONAF, Chile, and dataset of captured nymphs, developmental stage, and infection status at an individual basis for each population in each season.
